# Lung cancer screening

**DOI:** 10.1038/sj.bjc.6605660

**Published:** 2010-04-27

**Authors:** U Pastorino

**Affiliations:** 1 Division of Thoracic Surgery, Istituto Nazionale Tumori, Via Venezian 1, 20133, Milan, Italy

**Keywords:** lung cancer, screening, early detection, spiral CT

## Abstract

Lung cancer is the primary cause of cancer mortality in developed countries. First diagnosis only when disease has already reached the metastatic phase is the main reason for failure in treatment. To this regard, although low-dose spiral computed tomography (CT) has proven to be effective in the early detection of lung cancer (providing both higher resectability and higher long-term survival rates), the capacity of annual CT screening to reduce lung cancer mortality in heavy smokers has yet to be demonstrated. Numerous ongoing large-scale randomised trials are under way in high-risk individuals with different study designs. The initial results should be available within the next 2 years.

## The magnitude of the problem

Lung cancer kills more than 1.3 million people every year (Peto *et al*, 1996), with figures registering a continual increase in the Far East in countries such as China (Yang *et al*, 2004). Improvements in clinical management have been modest over the past 20 years, with an overall 5-year survival rate just above 10% in Europe (Verdecchia *et al*, 2007). Presence of metastatic disease at diagnosis is the main reason for treatment failure, and 5-year survival of patients resected in stage Ia is >70% (Goldstraw *et al*, 2007). In developed countries, smoking sanctions have achieved a significant reduction in the prevalence of active smokers and lung cancer mortality in males, but not yet in females (Levi *et al*, 2003). Despite the success of early prevention, a large cohort of former smokers remain at high risk of cancer for many years.

## Previous research involving other modalities

Early detection trials with chest radiography (CXR) and sputum cytology, funded by the US National Cancer Institute in the 1970s, were ineffective in decreasing lung cancer mortality, despite the higher proportion of early-stage cancer identified through screening (Melamed *et al*, 1984). Quite unexpectedly, the 25-year follow-up of the Mayo trial showed that overall mortality was higher in the CXR arm compared with the standard care arm (difference not reaching statistical significance, *P*=0.09), even though the survival rate of lung cancer patients diagnosed at an early stage in the CXR arm was much higher (69 *vs* 54% at 5 years, median 16 years *vs* 5 years, respectively) (Marcus *et al*, 2006). These results confirmed the inefficacy of CXR monitoring, as well as the occurrence of overdiagnosis in the intervention arm.

## Observational studies with low-dose spiral CT

The advent of low-dose spiral chest computed tomography (LDCT) opened a new perspective for early diagnosis, and initial studies conducted in Japan in the 1990s demonstrated the potential value of LDCT for lung cancer screening (Kaneko *et al*, 1996). Since then, the rapid technological development in multislice machines has improved the sensitivity and reliability of spiral CT, providing the concrete possibility of detecting pulmonary lesions of 3–4 mm in size in a few seconds, without the use of intravenous contrast.

In 1999, the Cornell University of New York published the first results of the Early Lung Cancer Action Project (ELCAP), showing that spiral CT scans had accuracy and sensitivity rates that were six times higher than CXR results in identifying very small lung tumours (56% <1 cm) with a resectability rate of 96% and a frequency of stage I neoplasms of 85%. This study provided solid guidelines for the management of CT-detected pulmonary lesions, including needle aspiration biopsy of small pulmonary lesions (Henschke *et al*, 1999).

The results of observational studies, including 64 475 subjects, are summarised with relative references in Table 1. The median age was 59 years (range 53–67), with minimum age ranging from 40 to 60 years. Five studies included non-smokers, representing 17–54% of participants in each trial, and an overall proportion of 20%. In the remaining eight studies, the median pack-years (p-y) was 43 years (range 30–45).

At baseline, the overall frequency of participants with suspicious non-calcified solid lesions was 20% (range 7–53), lung cancer detection rate was 1% (range 0.4–2.7), and the proportion of stage I lung cancer was 81% (range 50–100%). The two extensive Japanese studies (Sone *et al*, 2001; Nawa *et al*, 2002) that included a significant proportion of non-smokers (38–54%) had the lowest frequency of baseline lesions (7–11%) and lung cancer detection rates (0.4–0.5%). On the other hand, the highest baseline detection rate (2.7%) reported by Henschke *et al* (1999) is mainly attributable to the accrual of heavier smokers above the age of 60 years (median 67 years, 45 p-y) and prolonged diagnostic workup of lesions detected at baseline (up to 2 years). In fact, in the more recent Korean study (Chong *et al*, 2005), in which nearly half of the 6406 participants were low risk and 23% were non-smokers, the lung cancer detection rate at baseline was only 0.4%. Interestingly, the use of the latest generation spiral CTs (8–16 slice) increased the frequency of subjects with suspicious nodules above 50%, but the lung cancer detection rate was not substantially greater (1.1%) (Veronesi *et al*, 2008). Ten studies reported the number of lung cancers detected at first annual CT repeat (Table 1): a total of 197 cases from 56 252 subjects, corresponding to a 0.3% rate (range 0.1–1.4) and a cumulative frequency at 2 years of 1.4% (range 0.6–2.6).

In studies in which the enrolled population demonstrated similar cancer risk factors, most of the differences observed in the baseline detection rate were related to the diagnostic algorithm, the analysis of CT images, and the definition of a positive screen applied by each centre, and tended to level up with the first CT repeat. In fact, excluding the two Japanese studies with a high proportion of non-smokers, the cumulative lung cancer detection rate by the second year of screening was 1.6% (range 1.3–2.6, Table 1).

## Randomised trials with low-dose spiral CT

A number of randomised trials are currently being performed worldwide, involving more than 90 000 individuals (Table 2).

Of particular interest is the Lung Screening Study (LSS), a completed feasibility trial carried out in the United States, which randomised 3318 smokers (age 55–74 years, ⩾30 p-y, median 54 p-y) to LDCT or CXR, performed at baseline and first year repeat (Gohagan *et al*, 2005). The LSS confirmed LDCT’s higher lung cancer detection rate compared with CXR: 1.8 *vs* 0.5% at baseline, and 2.4 *vs* 1.3% at the first year repeat. On the basis of the success of LSS, the National Cancer Institute, in collaboration with the American College of Radiology Imaging Network, launched the National Lung Screening Trial, which represents the largest randomised controlled trial comparing LDCT with CXR, with lung cancer mortality as the end point (Clark *et al*, 2008). More than 53 000 participants were enrolled from over 30 centres across the United States. The National Lung Screening Trial has 90% statistical power to detect a 20% reduction in lung cancer mortality in the LDCT arm. The trial design included three annual screening examinations, which were completed in 2006, and subjects are currently in follow-up, with final results expected in 2011.

The European screening programme currently includes six randomised studies: the NELSON trial in the Netherlands, the Danish Lung Cancer Screening Trial in Denmark, The LUSI trial in Germany, and three Italian studies, namely, DANTE-Milan and ITALUNG-Florence, and MILD-Milan, all comparing annual CTs with observation without screening.

The NELSON trial was designed to detect a 25% reduction in lung cancer mortality in subjects aged 50–74 years having smoked >15 cigarettes per day for >25 years, or >10 cigarettes per day for >30 years, with a planned population of 20 000 subjects and three 16-slice CT examinations at years 1, 2, and 4 in the screening arm (van den Bergh *et al*, 2008). The NELSON trial accrued nearly 16 000 individuals in the Netherlands and Belgium, and the remaining 4000 needed to reach statistical power were recruited from Denmark. Initial results of Nelson have shown a 0.9% lung cancer detection rate at baseline, with a 99.9% negative predictive value of the diagnostic algorithm, based on the automated assessment of volume and doubling time (van Klaveren *et al*, 2009). The proportion of invasive procedures that revealed benign disease was 27.2%. The Danish Lung Cancer Screening Trial has a similar risk population (50–70 years, ⩾20 p-y), but includes annual CT screening for 5 years, with results regularly uploaded into the Nelson database. The baseline lung cancer detection rate of the Danish Lung Cancer Screening Trial was similar to the one of Nelson (0.8%) (Pedersen *et al*, 2009).

A similar trial, LUSI, was launched in Germany in 2007, and is now in the randomisation phase (Becker *et al*, 2008).

The DANTE-Milan study was the first European randomised trial to be launched in 2001, enrolling 2472 subjects (60–74 years, ⩾20 p-y) in annual CT screenings or in observation for 4 years (Infante *et al*, 2008). At baseline, participants in both arms had to undergo one single CXR and sputum cytology examination, which led to lung cancer detection in eight patients in the control arm. The ITALUNG-Florence randomised 3206 subjects (55–69 years, ⩾20 p-y) to annual CT *vs* observation for 4 years. Baseline CT detected 21 lung cancers in 20 subjects (1.5%) (Lopes Pegna *et al*, 2009). The Multicentric Italian Lung Detection trial started in Milan in 2005 and is still open to accrual. Until now, more than 4400 participants have been assigned to LDCT or control, for a period of 10 years. In the LDCT arm, a second randomisation has been planned to compare yearly CT screening with testing every 2 years. All participants will undergo an antitobacco counselling programme, pulmonary function test evaluation, and tissue sampling for extensive biomarker assessment.

A pilot trial, entitled Depiscan, was launched in France in 2002, to assess the feasibility of a screening programme with accrual based on 232 general practitioners and occupational physicians. The trial entered only 621 subjects in 2 years, and was closed because of insufficient accrual (41% active investigators) and poor compliance (Blanchon *et al*, 2007). At baseline, eight lung cancers (2.4%) were detected in the CT arm and one (0.4%) in the CXR arm. A further randomised trial is still in the planning phase in the United Kingdom.

The DANTE study is the only European study with published results on screening efficacy (Infante *et al*, 2009). After a median follow-up of 3 years, lung cancer was detected in 60 (4.7%) subjects receiving LDCT and in 34 (2.8%) controls (*P*=0.016), with a higher percentage of stage I lung cancer (54 *vs* 34%, respectively; not significantly different: *P*=0.06), corresponding to a three-fold increase in the total number of stage I lung cancer cases (33 *vs* 12; *P*=0.004). However, the number of stage III–IV lung cancers was not reduced in the LDCT arm (24 *vs* 21 cases), and lung cancer mortality was identical in the two arms (20 deaths each), as well as mortality due to other causes (26 *vs* 25). In summary, the mid-term results of the first randomised trial have not confirmed LDCT screening as having a measurable effect in diminishing lung cancer mortality. Nonetheless, a definitive answer regarding the concrete possibility of preventing mortality in heavy smokers with annual LDCT screening will be determined by the long-term follow-up of all randomised trials, the first of which is the National Lung Screening Trial. A research priority for the coming years will be to guarantee that all European trials, in which the control arm is observational and intervention is prolonged beyond 3 years, can be pooled in a large meta-analysis, to reach adequate statistical power.

## Issues specific to screening with spiral CT

### Prevalence of false-positive findings

The frequency of non-calcified pulmonary lesions in heavy smokers aged ⩾50 years is related to the sensitivity of the spiral CT: with the 16-slice CT, the lung cancer diagnostic rate is about 1% per year, but the probability of detecting benign lesions is 50 times higher (Veronesi *et al*, 2008, Table 1). Consequently, the overall screening performance depends on the selected diagnostic algorithm.

In the Mayo CT trial, the cumulative frequency of subjects with suspicious lesions was 74% at 5 years, but only 6% of them proved to have cancer, with a 70% rate of false-positive findings among all participants in the study (Swensen *et al*, 2005), whereas in the Milan study, using single-slice CT and a cutoff diameter above 5 mm, the frequency of subjects with suspicious lesions was only 19% at 5 years, 20% of whom proved to have cancer, with only 15% false-positive CTs overall (Pastorino, 2006). Nonetheless, the cumulative lung cancer detection rate was 4% in both trials (average 0.8% per year), with other major end points such as resectability and proportion of stage I disease being very similar. These results are intriguing in terms of screening performance, as one would expect a more sensitive diagnostic workup to be associated with a better clinical outcome.

Diagnostic assessment of small pulmonary nodules requires serial CT scanning for the detection of morphological changes, and the availability of reliable software for fully automated three-dimensional segmentation and highly consistent volume measurement of lung nodules has become essential in evaluating growth as evidence of potential malignancy (Wiemker *et al*, 2005; Marchianò *et al*, 2009).

Long-term follow-up of lesions ⩽5 mm suggests that these nodules require no additional workup. For lesions between 5 and 10 mm, surveillance of growth should be performed by three-dimensional volumetry, as volume doubling is equivalent to only a 26% increase in diameter, a difference that may be difficult to detect with manually guided diameter measurements. Changes in size indicative of a doubling time ranging from 30 to 360 days are consistent with cancer and require further investigation (Libby *et al*, 2004).

Pure non-solid lesions, or ground-glass opacities, have a low risk of malignancy (10–15%), usually represented by well-differentiated bronchioloalveolar carcinomas (BACs), but their volume and growth are more difficult to evaluate. Part-solid lesions have a higher risk of malignancy (up to 50%), directly related to the size and growth of the solid component (Henschke *et al*, 2002).

### Role of PET in diagnostic evaluation of positive CT screens

Large meta-analyses have demonstrated the clinical value of PET in the differential diagnosis of undetermined pulmonary nodules detected by spiral CT, with a sensitivity rate of 96–97%, a specificity of 78–82%, and an accuracy rate reaching 92% with the CT/PET fusion machine (Kim *et al*, 2007). Moreover, a randomised trial demonstrated that preoperative PET significantly improves lung cancer staging, by detecting occult metastases in otherwise resectable patients (van Tinteren *et al*, 2002).

In a recent paper, we reported that selective use of PET scans may be helpful in the management of CT-detected lesions ⩾7 mm. In the first 5 years of screening, PET was applied to only 1.4% of spiral CTs, with an overall sensitivity rate of 94%, specificity of 82%, and an accuracy rate of 88% (Pastorino *et al*, 2009). In addition, we demonstrated that the intensity of metabolic activity, expressed by standardised uptake value (SUV), can predict long-term survival in screening-detected lung cancer in a non-invasive manner. In fact, 5-year survival was 100% for SUV ⩽2.5, 60% for SUV >2.5 and <8, and only 20% for SUV ⩾8 (*P*=0.001). If confirmed by other studies, these results might improve the clinical management of CT-detected tumours in the future, reducing the risk of unnecessary treatment for indolent disease.

### Overdiagnosis of indolent tumours

Detection and treatment of slow-growing disease, also called overdiagnosis, represents one of the most likely explanations for the lack of mortality reduction observed in chest X-ray-screening trials, despite a significant increase in the overall resectability rate and proportion of stage I cancers in the intervention arm (Marcus *et al*, 2006). Overdiagnosis bias refers to the screening-related detection of cancers that would not have contributed to the death of the patient because of competing causes of death or because the lesion is indolent. The occurrence of slow-growing lung cancer has often been rejected because of similar histopathological and molecular features of screening and non-screening-detected cases. However, the significant rise in the frequency of adenocarcinoma from 32% of all cases in the SEER database from 1983 to 1987 (Travis *et al*, 1995) to more than 70% (range 55–95%) in CT-screening trials (Table 1 references) may well represent overdiagnosis. An international panel has reviewed the histopathological features of 279 stage IA adenocarcinomas belonging to the I-ELCAP database, to determine whether survival differed from the proportion of the BAC component (Vazquez *et al*, 2008). The revision confirmed that 81% of CT-detected adenocarcinomas show a substantial BAC component, and that 5-year survival was 100% for pure BAC and 95% for mixed BAC. Such a sudden shift towards adenocarcinoma and BAC cannot be explained by major changes in the epidemiology of tobacco consumption, and is strongly suggestive of an excessive detection of indolent disease by CT screening.

### Morbidity of CT screening

The side effects and morbidity of CT screening are difficult to assess using observational studies. The frequency of surgical procedures for benign disease, not including bronchoscopy and fine-needle aspiration biopsy, is reported in Table 3. Invasive biopsies for benign lesions represent, on an average, 18% (range 0–33%) of all surgical procedures and 0.3% of individuals undergoing CT screening. Postoperative mortality is not mentioned in most studies, and numbers are too low for a proper estimate of the risk. Randomised trials may allow a better evaluation of the impact of CT screening on quality of life and proportion of unnecessary invasive diagnostic procedures and hospital admissions, including morbidity and mortality, by comparing the two arms.

### Lung cancer mortality and survival in CT screening

In 2005, Swensen *et al*, comparing the lung cancer mortality rates in the Mayo CT study with those observed in the previous CXR trial, found no differences in lung cancer mortality rates between the two studies in the subset of men aged 50 years or older (2.8 *vs* 2.0 per 1000 person-years) (Swensen *et al*, 2005). On the other hand, in 2006, the International ELCAP study group published a report on the efficacy of CT screening, in which the overall survival of 484 screening-detected lung cancer patients was 80% at 10 years, regardless of stage and treatment, and reached 92% in the subset of clinical stage I resected within 1 month of diagnosis (I-ELCAP, 2006). The authors therefore concluded that CT screening could prevent 80% of all lung cancer deaths in high-risk individuals. However, the analysis was focused only on cancer detected at baseline and first CT repeat, without long-term follow-up of the entire cohort. Moreover, patients’ outcome at 10 years was projected from a median observation period of only 3 years, and lung cancer-specific survival was used instead of overall survival.

A concurrent meta-analysis of three single-arm studies (Mayo Clinic, Milan and Lee Moffitt Cancer Center) including 3246 subjects and 10 942 person-years was performed by the epidemiologists of Memorial Sloan Kettering Cancer Centre, using a validated model to predict lung cancer incidence and mortality (Bach *et al*, 2007). The results of this study, published in JAMA, 2007, demonstrated that CT screening resulted in a 3.5-fold increase in lung cancer diagnoses and a 10-fold increase in surgical resections. However, the number of cases of advanced lung cancer and lung cancer death did not differ from expected numbers of advanced lung cancer and lung cancer death, with those numbers generated from a simulation model that used the CARET data. Quite interestingly, the results were consistent among all three centres, despite the potential differences in epidemiological risk and screening methodology, mirroring the findings of the only published randomised trial, the DANTE study, in which lung cancer mortality was identical in the CT screening and observational arms (Infante *et al*, 2009). Why was earlier detection in the first 2 years of screening not effective in preventing, at least to some extent, the occurrence of metastatic lung cancer in the long term? One possible explanation is that spiral CT is unable to intercept the fast-growing or ‘most aggressive’ lung cancers before the onset of metastases.

## Role of biomarkers in lung cancer screening

Biomarker research in CT screening trials, combining non-invasive genomic and proteomic analysis of target tissues, may represent a significant improvement in early detection, offering a potential contribution to diagnostic algorithms, assessment of individual risk, as well as management of CT-detected cancers. The issue of early detection biomarkers in lung cancer has recently been reviewed by Montuenga and Pio (2009), with a focus on blood and sputum evaluation. Even though there is no single validated molecular biomarker for early lung cancer detection, a complex spectrum of molecular profiles in body fluids of subjects enrolled in CT screening trials now offers truly innovative prospects for early diagnosis.

For instance, proteomic analysis of plasma, using SELDI-TOF and protein chip technology, has revealed high sensitivity and specificity, with an 8.7-fold excess risk of cancer for CT-screened individuals with elevated SAA protein levels (Cremona *et al*, 2010). Another new field of research is represented by breathing analysis, using electronic sensors (e-nose) to capture volatile compounds, or to extract genomic and proteomic material from frozen exhaled breath condensation (Horváth *et al*, 2009).

## Conclusions

Prospective randomised controlled trials remain the most appropriate instrument to test the efficacy of CT screening in heavy smokers. The ongoing randomised studies currently being conducted around the world have the adequate size and appropriate design to provide unequivocal evidence of even modest reductions in lung cancer mortality. In the meantime, CT screening for lung cancer should be considered as an experimental procedure, which is not to be offered or promoted outside controlled clinical trials. Innovative strategies could be tested to implement early detection research, possibly combining radiological monitoring with pharmacological intervention on smoking cessation, and the validation of lung biomarkers.

## Figures and Tables

**Table 1:** Lung cancer CT screening: results of observational studies

						**Lung cancers**		
	**Subjects**	**Age (years)** a	**NSM** b	**p-y** c	**CT lesions** d	**Baseline** e	**Stage I** f	**First repeat** g
Henschke *et al*, 1999	1000	67	0	45	233 (23)	27 (2.7)	85	—
Sone *et al*, 2001	5483	64	54	—	588 (11)	23 (0.4)	100	27 (0.5)
Nawa *et al*, 2002	7956	56	38	—	541 (7)	36 (0.5)	78	4 (0.1)
Sobue *et al*, 2002	1611	59	14	—	186 (12)	14 (0.9)	71	22 (1.4)
Diederich *et al*, 2004	817	53	0	45	350 (43)	12 (1.5)	64	—
Swensen *et al*, 2003	1520	59	0	45	780 (51)	27 (1.7)	74	13 (0.9)
Pastorino *et al*, 2003	1035	58	0	40	199 (19)	11 (1.1)	55	11 (1.1)
Bastarrika *et al*, 2005	911	55	0	30	291 (32)	12 (1.3)	83	2 (0.2)
Chong *et al*, 2005	6406	55	23	—	2,255 (35)	23 (0.4)	56	—
Novello *et al*, 2005	519	59	0	—	241 (47)	5 (1.0)	67	3 (0.6)
MacRedmond *et al*, 2006	449	55	0	45	111 (25)	2 (0.4)	50	4 (0.9)
I-ELCAP 2006	31 567	61	17	30	4186 (13)	410 (1.3)	85	74 (0.2)
Veronesi *et al*, 2008	5201	58	0	44	2754 (53)	55 (1.1)	66	37 (0.7)
								
Overall	64 475	59	20	—	12 715 (20)	657 (1.0)	81	197 (0.3)

Abbreviations: CT=computed tomography; NSM=non-smokers; p-y=pack-years.

a Median age of participants.

b Proportion of non-smokers.

c Median pack-years.

d Subjects with suspicious non-calcified solid lesions (percentage of participants).

e Lung cancers detected at baseline (percentage of participants).

f Percentage of lung cancers detected in stage I at baseline.

g Lung cancers detected at first annual CT repeat.

**Table 2:** Lung cancer CT screening: randomised studies

**Study**	**Country**	**Study design**	**Year started**	**Subjects enrolled**	**Age range (years)**	**Years screen**
LSS (Gohagan *et al*, 2005)	USA	CT *vs* CXR	2000	3318	55–74	2
DANTE (Infante *et al*, 2008)	Italy	CT *vs* obs	2001	2472	60–74	4
NLST (Clark *et al*, 2008)	USA	CT *vs* CXR	2002	53 000	55–74	3
NELSON (van den Bergh *et al*, 2008)	NL–B	CT *vs* obs	2003	15 822	50–74	5
ITALUNG (Lopes Pegna *et al*, 2009)	Italy	CT *vs* obs	2004	3206	55–69	5
DLCST (Pedersen *et al*, 2009)	DK	CT *vs* obs	2004	4104	50–70	5
MILD (Pastorino *et al*, 2006)	Italy	CT *vs* obs	2005	4479	49–75	10
LUSI (Becker *et al*, 2008)	Germany	CT *vs* obs	2007	4000	50–69	5

Abbreviation: CT=computed tomography; CXR=chest X-ray; DLCST=Danish Lung Cancer Screening Trial; LSS=Lung Screening Study; MILD=Multicentric Italian Lung Detection; NL–BM=the Netherlands and Belgium; NLST=National Lung Screening Trial; obs=observational studies.

**Table 3:** Lung cancer computed tomography screening: surgical procedures for benign disease

		**Number of surgical procedures**			
	**Subjects**	**Total**	**Cancer**	**Benign**	**Benign disease (%)**
Henschke *et al*, 1999	1000	28	27	1	4
Sone *et al*, 2001	5483	72	56	16	22
Nawa *et al*, 2002	7956	58	40	18	31
Sobue *et al*, 2002	1611	36	31	5	14
Diederich *et al*, 2004	817	14	11	3	21
Swensen *et al*, 2003	1520	83	68	15	18
Pastorino *et al*, 2003	1035	43	36	7	16
Bastarrika *et al*, 2005	911	14	14	0	0
Novello *et al*, 2005	519	14	12	2	14
MacRedmond *et al*, 2006	449	9	6	3	33
Veronesi *et al*, 2008	5201	107	92	15	14
					
Overall	26 502	478	393	85	18
